# Framing the future of food: The contested promises of alternative proteins

**DOI:** 10.1177/2514848619827009

**Published:** 2019-02-06

**Authors:** Alexandra E Sexton, Tara Garnett, Jamie Lorimer

**Affiliations:** University of Oxford, UK

**Keywords:** Alternative proteins, cultured meat, promise, livestock, good food

## Abstract

This paper offers a critical examination of the narrative landscape that has emerged with a new movement of alternative proteins intended as substitutes for conventional meat, milk and other animal-based food products. The alternative protein approaches analysed include edible insects, plant-based proteins and cellular agriculture, the latter of which encompasses ‘cultured’ or ‘clean’ meat, milk and egg products produced in vitro via cell-science methods. We build on previous research that has analysed the promissory narratives specific to cultured/clean meat by examining the key promises that have worked across the broader alternative protein movement. In doing so, we develop a five-fold typology that outlines the distinct yet interconnected claims that have operated in alternative protein promotional discourses to date. The second part of the paper examines the counter-narratives that have emerged in response to alternative protein claims from different stakeholders linked to conventional livestock production. We offer a second typology of three counter-narratives that have so far characterised these responses. Through mapping this narrative landscape, we show how different types of ‘goodness’ have been ascribed by alternative protein and conventional livestock stakeholders to their respective approaches. Moreover, our analysis reveals a series of tensions underpinning these contested food futures, many of which have long histories in broader debates over what constitutes better (protein) food production and consumption. The paper's discussion contributes to ongoing research across the social sciences on the ontological politics of (good) food, and the key role of narratives in constructing and contesting visions of ‘better’ food futures.

## Introduction

The central role played by narratives in shaping how and what we eat has been convincingly argued across social-science research on food (Eden, 2011; Evans and Miele, 2012; Goodman, 2004). The narratives typically analysed in this research are those crafted by food producers and retailers, and are disseminated through the textual and visual language of food labels and other forms of advertising across in-store and online media. Their primary aim is often intended as a ‘knowledge fix’ (Eden, 2011) to help consumers make more informed and ultimately better food choices. Increasingly, this fix has involved attempts to (re)connect food consumers with food producers by making visible the actors and processes involved in food networks (Cook et al., 2004), and in doing so encourage us to cultivate more caring relationships with the ‘distant strangers’ behind our everyday eating (Corbridge, 1998, cited by Goodman, 2004: 893). Food narratives can also evoke and invent more compassionate human–animal relationships (Miele, 2011), as well as stigmatise and revalorise entire dietary lifestyles, such as veganism (Doyle, 2016). Despite their influential role, however, food narratives can often fail in their intended purpose, instead causing confusion and even indifference on account of the sheer volume and contradictory nature of information that modern consumers must navigate within their everyday foodscapes (Eden et al., 2008).

The aim of this paper is to examine the narratives deployed by a new movement of producers and advocates of ‘animal-free’ alternatives.^1^ These products have attracted multibillion-dollar investments from some of the biggest names in global business, including Bill Gates and Richard Branson. Over the last decade, key promoters of these products have consistently driven visions across industry and popular media of what the future of food will look like (Carrington, 2018; Chu, 2017). The products can be organised into three distinct categories of alternative: plant-based proteins, edible insects, and a group referred to as ‘cellular agriculture’.^2^ This latter group encompasses products commonly referred to as ‘cultured’ or ‘clean’ meat, milk and other animal products, created either through culturing stem cells outside (in vitro) animal bodies, or through the genetic modification and fermentation of yeast cells.^3^ While a small proportion of this activity has been driven by university-based research, the majority of these ventures have involved private companies, especially the business models of the Big Tech start-up region of Silicon Valley, California. A collective nomenclature for these foodstuffs is still in flux (see Stephens et al., 2018 for extended discussion). The term ‘alternative proteins’ has been recently adopted within the sector and in related writings (e.g. Carrington, 2018; Chu, 2017). In this paper, we collectively refer to these products as APs, and also as ‘the movement’ in line with other academic writings (e.g. Mouat and Prince, 2018). Our use of this latter term is intended to reflect the sense of collective momentum over the last 5–10 years, particularly within Anglophone regions, that has seen a flurry of academic and entrepreneurial interest in developing alternative approaches to conventional livestock products. This is not to suggest, however, that the AP communities we discuss necessarily and always self-identity as a unified movement. There are instances in which the enactment of a unified community has been welcomed and institutionally endorsed through industry conferences and research grant programs.^4^ Yet there have also been distinct efforts within this community to distinguish their respective endeavours: for example, some proponents of cellular agriculture and plant-based products have explicitly tried to distance themselves from insect-based approaches, often citing the ethical ambiguity of slaughtering insects for food. We thus use the term ‘AP movement’ with awareness of these internal tensions and the continued instability of its associated terminology.

All of the APs we discuss have a degree of precedency: insects and plant-based foods are not entirely novel in the history of human consumption (DeFoliart, 1995; Shurtleff and Aoyagi, 2013), nor are cellular approaches to edible protein production (e.g. single-cell proteins, Quorn).^5^ Yet despite these histories, AP narratives have noticeably cultivated a sense of breaking from what has gone before. As such, their promises have worked collectively to create a vision of APs as a paradigmatic shift in protein production and consumption, through which they make the ultimate promise of a *better* future food system.

Due to the relative infancy of the sector, many of these APs have been consumed more as narratives than as tangible, eat-able foodstuffs (Mouat and Prince, 2018). There are currently no cellular agriculture products on the market, and while a number of plant-based and insect AP companies have launched products they have until very recently remained within a small number of countries and specialist retailers. Many of these products have relatively high price points.^6^ To create consumer demand and to gain investment capital, AP narratives have so far been characterised by a series of promises of what they *will* achieve once on the market, and how they will improve the production and consumption of protein foods. We adopt the term ‘promissory narratives’ to capture this characteristic, a conceptual tool borrowed from writings on the sociology of expectations (Brown and Michael, 2003). The value of this concept is that it takes seriously the promises surrounding (food) innovations and their role in summoning particular futures to do important political and material work in the present, such as generating consumer interest and raising venture capital (Brown, 2003). A number of recent studies have adopted this approach to consider the framings specific to cultured meat (Chiles, 2013; Jönsson, 2016; Stephens, 2013). In this paper, we extend this research by considering the promissory narratives at work across the broader pantheon of AP products that have emerged over the last decade, including cultured milk and eggs, plant-based proteins and insect products. To our knowledge, no research has mapped the multiple promises made by this broader AP *movement*, rather than in relation to individual products.

Through an empirical analysis of the promissory narratives employed by leading AP stakeholders, we develop a typology of five key promises. We title these: ‘Healthier bodies’, ‘Feeding the world’, ‘Good for animals and the environment’, ‘Control for sale’ and ‘Tastes like animal’. We show that these promises work collectively to diagnose a series of problems relating to current livestock production, and to present APs as the effective, logical solutions. Such narratives can be seen to (re)produce the rise of protein as a particular food-related concern across multiple fronts, from the health of individuals to securing a hunger-free and climate-stable future.^7^ It is important to note, however, that while the paper sets out distinct promissory categories, each of the promises we identify are often themselves made up of multiple framings that coexist and overlap, and are sometimes operationalised within different contexts (i.e. to speak about different AP products and appeal to different problems and audiences).

These products enter an already crowded and contested discussion as to how best to deliver a better food system, and specifically what role (if any) livestock should play (Garnett, 2015). As a consequence, there has been a backlash against, and appropriation of, AP narratives by different stakeholders in the current livestock sector in recent years. These developments have yet to be examined in existing AP research. The second part of the paper begins this work by identifying the three key counter-narratives that characterise the livestock industry's response to APs. In doing so, we reveal a series of tensions around which these contested food futures have coalesced, many of which we show in the final section of the paper have long histories in broader debates over what constitutes better food production and consumption. In mapping these contested narratives, the paper builds on our recent work examining how APs are being constructed as ‘food’ by their developers and ‘non-food’ by their detractors (Sexton, 2016, 2018). It also contributes to recent discussions elsewhere on the politics of framing in contemporary foodscapes (Bryant and Goodman, 2004; Evans and Miele, 2012; Goodman, 2004), on the ontological multiplicities of (animal-based) food (Yates-Doerr, 2015a), and in the construction and contestation of food-system futures (Jarosz, 2011, 2014; Margulis, 2013).

## Methodological approach

Promissory narratives have proven a valuable conceptual lens for the study of technological innovations (Brown, 2003; Brown and Michael, 2003), and have been a key focus in recent analyses of cultured meat (Jönsson, 2016; Mouat and Prince, 2018; Stephens, 2013). As with other innovations, these studies have shown the diverse political, economic and ontological functions of AP narratives and the particular audiences they have attempted to engage. Examples include the recruitment of intellectual power from scientific communities (Stephens, 2013), the creation of prospective markets to attract investors (Mouat and Prince, 2018), and the stabilisation of APs as safe yet exciting, familiar yet progressive, and edible yet novel foods (Jönsson, 2016; Sexton, 2018; Stephens, 2013). These studies have also shown the broad variety of textual media involved in the promissory landscape of APs. This ranges from product packaging and company websites (Sexton, 2016), to photographs (Stephens and Ruivenkamp, 2016), and infographics (Mouat and Prince, 2018). Key promissory work is also done by physical proofs-of-concept, such as the cultured beef burger in 2013 (Post, 2012; Stephens and Ruivenkamp, 2016) and a number of speculative design experiments (Catts and Zurr, 2002; Next Nature Network, 2014).

To extend our analysis beyond cultured meat, we identified the shared promises at work across the different approaches of the AP movement. We selected 12 key AP stakeholders (10 start-ups and 2 non-profit advocacy organisations) for analysis, all of whom have been identified by the movement itself and in media coverage as the key influencers of this emerging sector (see Table 1 for expanded details). This selection process and our data analysis were informed by 30 semi-structured interviews conducted by the first author with company founders, employees, investors, third-sector advocacy groups and other key stakeholders working in the AP movement between 2014 and 2016. The interviewees included representatives from all of the stakeholders listed in Table 1 except two, one of which was due to recruitment issues and the other had not been founded during the fieldwork period. The interviews were conducted in Europe and the US, predominantly within the San Francisco Bay Area (aka ‘Silicon Valley’) but also in Los Angeles and New York City. As with the stakeholders listed in Table 1, the interviewees were largely selected by those identified as industry leaders within the three AP sub-categories of cellular agriculture, edible insects and plant-based analogues. Their status as leaders was typically due to the amount of venture capital and media hype they had raised to date, the scope of their partnerships with the established food sector, and their development of specific production methods and product types. The recruitment strategy involved initial contact through e-mail and phone, as well as face-to-face meetings at industry events; this sampling was snowballed out via recommendations made by interviewees as the research progressed. The response rate was relatively high, an outcome that Author 1 attributes to the timing of the research coinciding with the relative infancy of the sector, thereby allowing greater access to individuals in elite positions (e.g. company founders). Where recruitment difficulties did occur, these were typically with investors, an outcome that is not uncommon in social science research on financial elites (Aguiar and Schneider, 2016; Desmond, 2004).

**Table 1.** List of selected AP stakeholders for narrative analysis.

AP stakeholder	AP approach	Location	Selection rationale
Beyond Meat	Plant-based (meat)	Los Angeles, CA	One of the early plant-based companies to launch in the recent AP movement; has raised $72million in funding rounds to date with high-profile investors including Bill Gates and Tyson Foods^8^
JUST (formerly Hampton Creek)	Plant-based (egg) Cellular agriculture (meat)	San Francisco Bay Area, CA	One of the early plant-based companies to launch in the recent AP movement, and first to expand to cultured meat; has raised $220 million in funding rounds to date with high-profile investors including Eduardo Saverin and Khosla Ventures
Impossible Foods	Plant-based (meat)	San Francisco Bay Area, CA	First AP venture to develop plant-based heme; has raised $387.5 million in funding rounds to date with high-profile investors including Bill Gates, Khosla Ventures and Li Ka-shing
Perfect Day Foods (formerly Muufri)	Cellular agriculture (milk)	San Francisco Bay Area, CA	The first cellular agriculture company developing milk
Clara Foods	Cellular agriculture (egg whites)	San Francisco Bay Area, CA	The first cellular agriculture company developing egg whites
Finless Foods	Cellular agriculture (fish)	San Francisco Bay Area, CA	The first US cellular agriculture company developing fish
Memphis Meats	Cellular agriculture (meat)	San Francisco Bay Area, CA	Highest-profile cultured meat company in US (at time of writing); has raised $17million in Series A funding round from investors including Bill Gates, Cargill and Richard Branson
Mosa Meats	Cellular agriculture (meat)	Maastricht, NL	Spin-off company of Professor Mark Post, creator of first cultured meat burger in 2013. First cultured meat company to launch in Europe
Exo	Insect (protein bars)	New York, US	One of the first US insect companies to launch products for human consumption; recently acquired by Aspire Food Group
Eat Grub	Insect (protein bars; whole insects)	London, UK	One of the first UK insect companies to launch products for human consumption
New Harvest	Non-profit advocacy group	New York, US	The first non-profit advocacy organisation specifically focussing on cellular agriculture; had direct role in founding a number of early cellular agriculture start-ups and remains a key player in industry-research-policy relations on cellular agriculture
Good Food Institute	Non-profit advocacy group with links to venture capital fund ‘New Crop Capital’	Washington, DC	Launched in 2016 and vocal advocate for the term ‘clean meat’, GFI is the second advocacy organisation in the US to focus on cellular agriculture. It also includes plant-based APs in its definition of ‘clean meat’. GFI is also connected with ‘New Crop Capital’, a venture capital fund with investments in a number of the ventures listed above. Along with New Harvest, GFI is a key player in industry-research-policy relations on cellular agriculture and plant-based proteins

Our analysis involved a close reading of the official websites and Instagram accounts of the AP stakeholders listed in Table 1. These media have served as core platforms for communicating information about APs to a variety of publics. For the websites, we focussed our sampling on the *Home* and *About Us* pages as these were considered key indicators of the messaging given most prominence by each stakeholder to promote both their mission as a brand and their product ranges.^9^ While other social media platforms are used by the AP movement, we focussed on Instagram given its rise as a particularly popular medium for food-related ventures to showcase their brands and establish more direct communication with their followers and customers (Eli et al., 2018; Lupton, 2016). Our sampling also included publicly available online media interviews and presentations (e.g. TEDTalks) given by our chosen stakeholders since 2013.^10^

To sample the counter-narratives, we conducted a search of media articles on Google News within the periods 2013–2014 and 2017–2018 using the search terms in Table 2.^11^ These searches produced hundreds of results, the majority of which were not specifically focussed on the counter-narratives of the conventional livestock industry. To refine our search, we included two further search terms: ‘response’ and ‘reaction’. This went some way to filtering out the more general reviews and news items on the AP movement, and made it easier to collect articles explicitly focussed on counter-narratives. We snowballed out from these initial results to find other links to news articles and supporting documents (e.g. petitions from industry lobby groups). While some material may have remained uncollected, we searched until we were confident we had saturated the dataset and identified a range of core thematic categories that we believe provide a useful first organisational map of recent counter-narratives to APs.

**Table 2.** Terms used for Google News search of counter-narratives.

Search terms for APs	Search terms for counter-narrative voices	Additional search terms
Alternative protein	Livestock farming	Response
Cultured meat	Livestock industry	Reaction
Clean meat	Farmers	
Insects		
Plant-based		

## A typology of promises

Good for people, animals and planet

– Clara Foods, *About Us* page

### Promise 1: Healthier bodies

**Figure 1.**
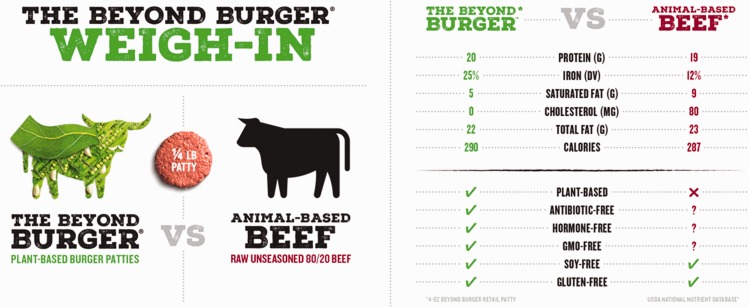
The Beyond Burger weigh in. Source: http://beyondmeat.com/about (accessed 7 June 2018).

The quote above captures the three stakeholder groups commonly identified by the AP movement as being harmed by conventional livestock systems, and for whom their products offer solutions. The primary way we encountered APs framed as being ‘good for people’ was through the promise of a healthier body.^12^ Most often this message was communicated through reference to a range of negative health impacts associated with conventional livestock products. Causal links were frequently made between animal foods and ‘chronic disease’, ‘health hazards’ and ‘nasty substances’. Sometimes specific statistics and illnesses were referenced – for example ‘16% cancer risk’ and ‘21% heart disease risk’.^13^ One way that APs promised consumers a healthier body was to position themselves as devoid of these health risks. Care was taken by AP stakeholders to emphasise their products as free of pathogens and contaminants, describing them as ‘cleaner’, ‘safer’, ‘disease-free’ and in one case ‘100% natural’ in comparison with their conventional counterparts. The absence of particular ingredients and production methods associated, sometimes explicitly, with industrially produced livestock also characterised these health messages. Figure 1 provides an example of this: here, we see the viewer invited to compare the nutritional profile of a conventionally produced beef burger with that of Beyond Meat's plant-based burger. Particular nutritional components are selected for this comparison including protein, cholesterol, iron, total and saturated fats, and calories. Except for calorie content, which is essentially the same, the alternative burger is claimed to perform more favourably in all the other categories. The absence of other already highly-coded elements like soya, antibiotics, hormones, GMOs, and gluten is also emphasised (cf. Sexton, 2016).

In contrast, the *presence* of favourable nutrients was also a key feature in the AP health messaging. Claims of being ‘nutrient-rich’, ‘nutritious’ and ‘nutritionally balanced’ were a common feature across the website and social media data. In addition to providing all-round nutrition, the quality and content of protein was specifically prioritised in the majority of AP narratives we analysed. Recurring themes included reassurances that the products were not only ‘high-protein’ but also offered a ‘complete protein source’ in that they contained ‘all the essential amino acids’ found in animal-sourced foods. This latter focus on offering *complete* protein was particularly salient in the insect narratives, a feature that Sexton (2018) has argued contributes to the familiarisation of insects as edible food to Western audiences. She states that claiming equivalent or in some instances greater levels of protein in comparison with conventional livestock meat is a key strategy not only for convincing Western consumers that insects are in fact edible, but also that they have more in common with livestock products than plant-based alternatives on account of the latter not always containing the full spectrum of amino acids and vitamins present in animal foods.

Yet it is important to note that not all insect products are being developed as direct replacements for meat in the sense of providing a like-for-like imitation of different cuts and products, such as chicken strips or beef burgers. Some recently founded companies are taking this route (see House, 2016), but the two ventures we review here have developed insect-based energy bars as their primary product. These products are not framed as meat but rather as ‘protein’. In this way, both these and other AP products join the recent nutricentric trend (Scrinis, 2008) that has seen protein treated as a food category in its own right. Examples we found across our data related not only to the AP products themselves (e.g. ‘the future of protein’) but also to the conventional animal foods they are intended to replace (e.g. ‘Fish is one of the healthiest proteins on the planet’).

The last decade has seen the emphasis on protein rise as a popular global food trend. The high protein content of everything from breakfast cereals to chocolate bars is now commonly highlighted as a desirable selling point, and protein supplements in the form of powders, liquids and energy bars have become a lucrative global market (Allied Market Research, 2017). The conceptualisation and promotion of protein as its own food group by the recent AP sector reflects these recent trends, but also follows previous narratives of plant-based diets: it continues the focus on protein as a strategy for dissolving the more obvious ontological boundaries between animal and non-animal foods by instead emphasising their respective, and crucially their *shared*, nutritional make-up (Sexton, 2018). Highlighting the presence of protein in plants serves to reorient culturally conditioned views of animal products as the best or only source of this nutrient, and thus in turn challenges the common practice of centring meals around animal products (Adams, 1990). Similar strategies have been adopted by the latest AP ventures, thereby allowing the categories of meat, dairy and eggs to be opened up further to non-conventional sources.

While the focus on protein by the latest APs may have disturbed the ontological categories of animal foods, it has done very little to disrupt the associations between this nutrient and notions of physical and cultural power. This is evident in the frequent descriptions of APs as ‘fuel’ and as products that can specifically help gym-goers and ‘adventurers’ ‘power’ through their activities. A number of the websites and social media accounts featured professional athletes and other active people as brand ambassadors, as well as infographics such as a muscly arm to communicate the product's protein content in grams. This visual language was most prominent in the media of the insect-based ventures, which is perhaps not surprising given their primary products are energy bars, a snack food that has risen in tandem with the broader trend and increased commercialisation of physical self-improvement (Bearne, 2018; Schlossberg, 2016). The emphasis on protein as a source of power thus appeals to the contemporary ideals of muscular and so-called ‘shredded’ aesthetics that have become aspirational body-types across genders (Maximus, 2018; Sifferlin, 2012). They have become attached to particular dietary trends such as clean eating and the Paleo diet. This focus also appeals to those who already partake in or aspire to a sense of adventure and a (re)connection with both nature ‘out there’ – an ideal that was reinforced through the prominent wilderness-landscape aesthetics in the promotional photography – and also with our ‘natural selves’ through a perceived return to cleaner, purer sources of protein.

### Promise 2: Feeding the world, now and forever

A narrative of feeding the world has been a consistent feature of the AP promissory landscape during the sector's relatively short history. Precisely who is being fed, with what products, and for what purposes has differed over time, although often in a way that allows multiple framings to coexist and overlap. One such framing situated APs within a global context, both in terms of the problems they claim to solve and the intended scale of consumption. These are products framed as meeting the challenge of an increasing global population with a growing demand for animal foods. In doing so, they will take a considerable share of the billion-dollar protein markets, whilst simultaneously addressing the consequences of the seemingly insatiable tastes for meat, dairy and eggs.

Two numbers often feature in these framings: ‘9 billion’ and ‘2050’. The first refers to the projected number of people expected to inhabit the planet, and the latter the year by which this total will be reached (Alexandratos and Bruinsma, 2012). These numerical abstractions depict an overcrowded, resource-strapped and specifically *protein*-deficient future in which the capacities of the planet cannot meet the demands of its population. Often in these Malthusian narratives the current system for producing protein – namely livestock farming – is framed as ‘broken’, ‘inefficient’ and ‘out-of-date’. APs are thus the Boserupian technological fix that will avert a nutritionally poor and pleasure-deprived future. As one company puts it on their website: ‘We make [our product] entirely from plants, without the destructive impact of livestock, so that you, your children, and your grandchildren's children will always be able to enjoy a good ol’ fashioned burger’. The ‘who’ being fed in these narratives thus extends across macro-timescales and geographies; it is both a global-level ‘us’ in the present, and our future descendants. In this sense, APs are framed as techno-cornucopias that will feed the world in a bountiful future in which hunger no longer exists, and the nostalgic pleasures and cultural value of animal foods have been preserved (Wurgaft, 2016; Yates-Doerr, 2015b).

Another number is often present in this narrative: ‘2 billion’ – referring to a more specific population, largely concentrated in the Global South, currently suffering from undernutrition and poverty (Patel, 2007). Within these narratives APs are framed explicitly as ‘food security solutions’ that promise both nutritional salvation and economic development for the hungry poor. Some of these visions appear to build on van der Weele and Tramper's (2014) early thought-experiment for cultured meat entitled ‘Every village its own factory?’. For example, the idea of installing ‘Cellular Agriculture Lifecycle pods’ (CALpods) in African refugee camps (Byrd, 2018), and JUST's recent collaboration with local producers in Liberia (Quinn, 2018) both envision AP production within local Southern contexts as a mechanism for providing low-cost, nutritionally rich and culturally tailored protein products.

### Promise 3: Good for animals and the environment

As well as being good for people, the environment and animals are two further beneficiaries identified in AP narratives. General statements of APs being ‘earth-friendly’, ‘eco-friendly’, ‘sustainable’ and creating a ‘smaller footprint’ were common. These were often contextualised within a broader narrative of an overstretched planet. Images of pastoral scenes, oceans and an iceberg accompanied statistics and statements concerning the ‘environmental devastation’ of conventional livestock and the benefits of the various AP approaches.

The planetary harms most frequently referenced were climate change, greenhouse gas emissions (GHGs), and the overuse of water and land. Often these harms were quantified – for example Beyond Meat's website displayed the following claims: ‘[we] currently use 70% of farm land for livestock’, ‘51% of GHG emissions driven by livestock rearing and processing’, and ‘29%: animal production share of agriculture water use’. The majority of these figures were cited without reference to their sources, and where references did appear they were often studies that have since been refuted for greatly over-estimating the impacts of livestock production, particularly their GHG emissions (e.g. Goodland and Anhang, 2011). In the insect cases, comparative breakdowns were given for the water usage and feed conversion (a signifier of land use) required to produce the same weight of crickets, chickens, pigs and cows.^14^ Impossible Food opted to focus on comparing individual-level impacts, with three infographics displaying the water saved (‘a 10-minute shower’), the GHGs avoided (‘18-miles worth of driving’) and the land spared for wildlife (‘75-square feet’) if their plant-based burger was chosen over a conventional beef burger.^15^ This strategy mirrors a recent publicity campaign by JUST which ran on the company's Instagram page and a number of billboards in New York City: the campaign included a series of images congratulating their customers for saving 2,097,203,366 gallons of water, preventing 7,783,289,850 grams of carbon emissions, and preserving 122,409,808 square-feet of land during 2015 as a result of buying their plant-based products. Such use of numerical abstractions to depict the inefficiency of livestock in converting environmental resources into calorie/protein output have clear echoes in the (neo)Malthusian narratives of the 20th century (e.g. Belasco, 1997).

In comparison with the promise of goodness for people and planet, those relating to animals were surprisingly less frequent in the media we reviewed.^16^ Of the references we did find, the *livestock* animal was evoked most commonly. The goodness promised by AP approaches to livestock animals typically centred on releasing them from the production process (e.g. ‘animal-free’, ‘100% vegan’), a consequence which was presented as creating a ‘kinder’ food system by removing the need for slaughtering and rearing animals in intensive systems. Only one company offered a quantitative framing of how livestock-animal welfare might be improved by citing data from the Food and Agriculture Organisation Corporate Statistical Database (FAOSTAT) on how many animals – ‘66 billion’ – are currently slaughtered for food every year.

### Promise 4: Control for sale

The notion that APs offer greater control in terms of the inputs and methods involved in production was central to the promises discussed in the previous sections. The historical framing of food products as the better, safer and overall more responsible choice on account of their technological and industrial modes of production has been similarly noted in other cases, including commercially produced white bread (Bobrow-Strain, 2008) and pasteurised cheese (Paxson, 2008). A key part of this messaging has been to offer the modern consumer what Bobrow-Strain (2008: 26) refers to as ‘control for sale’, whereby the relocation of production into the spaces of technoscience is seen as safeguarding against the uncleanliness and risks associated with processes that are seemingly ‘closer’ to nature. In the cases of the rise of industrial bread and pasteurised cheese, it was home-baking and small-scale artisanal production that were constructed as the risky spaces and practices to which industrial processes offered a safer alternative. In the case of APs, it was the bodies of animals, the landscapes within which they are reared, and the processes that turn their bodies into food – particularly ‘intensive’, ‘industrial’ systems – that were most frequently highlighted for their riskiness, both in terms of food safety and moral impurity (Douglas, 1966). The promise of control in the AP narratives was linked to the sector's relocation of production away from spaces and processes associated with specifically animal natures: for example cellular agriculture products were framed as ‘safer’ and ‘purer’ on account of their production within the ‘safe, sterile, controlled conditions’ of laboratories. Another company talked of ‘bypassing the ocean and fish farming’ and instead producing fish ‘in a clean, safe way *on shore*’ (our emphasis).

For cellular agriculture specifically, a secondary possibility of control was attributed to its technological methods. By removing the animal – and in doing so the perceived limits of Nature – and building products molecularly from the bottom up, many cellular agriculture advocates highlighted the opportunities for creating new and improved functional properties. Claims of ‘ingredients that deliver’ and ‘increased performance: fluffier meringues and lighter cakes’ appeared on cellular agriculture websites alongside promises of improvements to health and sustainability. Moreover, the ability to provide complete consistency and reliability in terms of quality and performance was repeatedly stressed. The promise of increased control afforded by the technoscience methods of APs thus represented a range of different types of goodness: *purity* in terms of food safety and ethical values; improved *healthiness* and *nutritional content* on account of building the products from the bottom up; expanded *culinary possibilities* for both the home baker and the commercial ingredients industry; and *consistency* in product quality, safety and performance that is contrasted with the unreliability of current agricultural systems reliant on ‘risky’ animal bodies.

### Promise 5: Tastes like animal

Despite the emphasis placed in AP narratives on their multiple planetary and individual benefits, AP developers have recognised that two common barriers to the uptake of animal-free alternatives, particularly amongst meat eaters, are a lack of familiarity and negative perceptions of their sensory properties (Hoek et al., 2011; Kenyon and Barker, 1998). The promise of offering the same taste, appearance and overall eating experience as conventional animal foods has consequently been a central characteristic of AP development. The eating experiences associated with animal foods are explicitly celebrated in the narratives of AP websites and social media. Statements such as ‘Eat what you love’, ‘We can't help it, we just love cheese!’ and ‘The revolutionary plant burger that looks, cooks, and satisfies like beef’ appeared in large bold letters on website homepages and Instagram posts, accompanied by longer product descriptions emphasising their ‘delicious’ taste and ‘mouth-watering juiciness and chew’.

Photographs and videos added to this display of sensory performance: on two of the plant-based company websites we found videos showing their burgers cooking on grills with close-up shots of the ‘meat’ spitting fat and turning from pink to brown as it cooked. One of the videos ended with the burger being dressed with bread buns, salad and mayonnaise, and shots of happy-looking eaters tucking in as the ‘meaty’ juices oozed on to their lips and hands. Visual content of APs in their retail packaging and being cooked and eaten made up the majority of the Instagram posts we reviewed. Much of this content included reposts of customer selfies with AP products, endorsements from celebrities and elite athletes, and advertisements for new product launches and stockists. Images celebrating popular holidays and cultural events such as national sports matches were also a common feature, particularly with the US plant-based companies. Often these involved the products being served up as the dishes typically associated with these events, such as a plant-based sweet potato pie for Thanksgiving and meat-free burgers ready for the ‘grilling season’.

**Figure 2.**
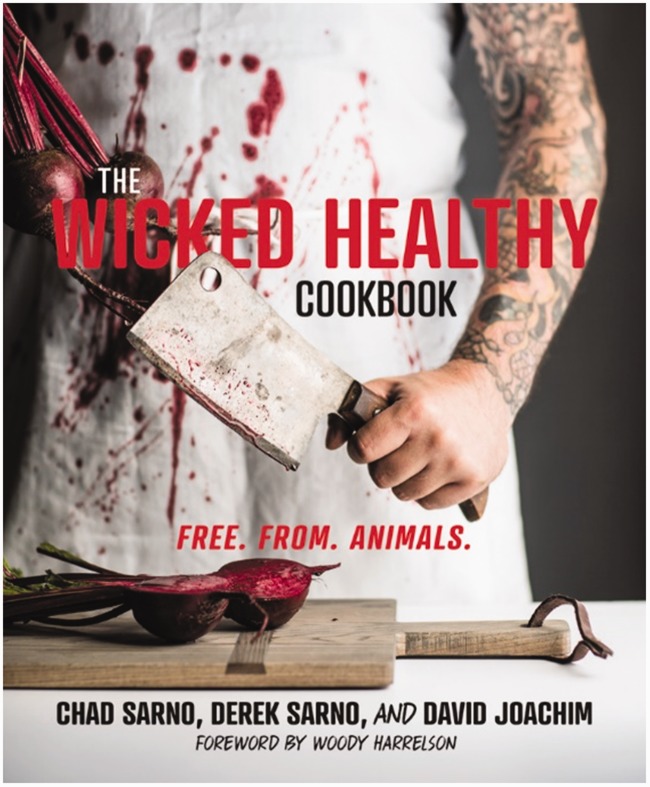
The Wicked Healthy cookbook. Source: www.wickedhealthyfood.com/wicked-healthy-cookbook/ (accessed 13 July 2018).

**Figure 3.**
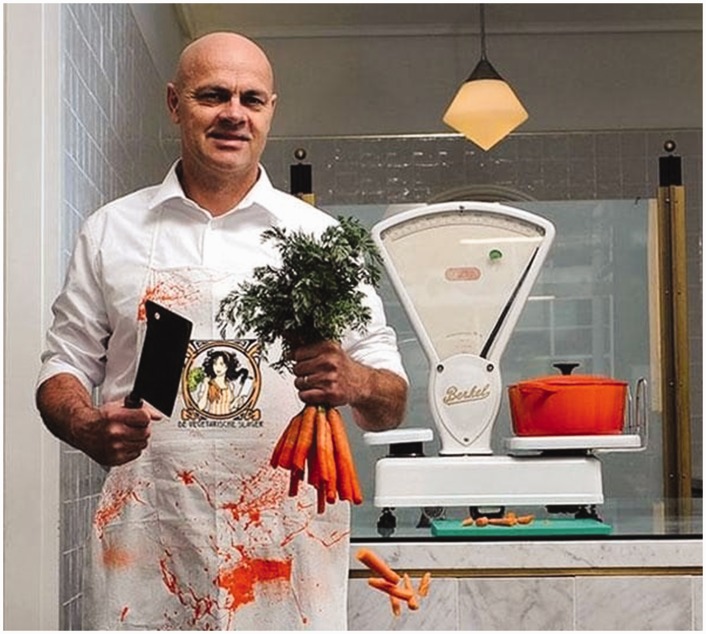
The vegetarian butcher. Source: www.telegraph.co.uk/food-and-drink/features/mock-meat-the-rise-of-the-vegetarian-butcher/ (accessed 13 July 2018).

As has been shown with other animal-free products, such strategies work to shift perceptions of animal-free eating from ‘dull to desirable’ (Doyle, 2016: 777), and accentuate that food which is good for us and the planet is also good to eat (Mol, 2009). This emphasis on pleasure was further highlighted in many of the Instagram posts where APs were often framed in the context of ‘treating’ oneself and were thus positioned as a guilt-free guilty pleasure. Such framings call to mind recent trends elsewhere that have worked to rebrand plant-based eating as a more hedonistic, visceral experience: these include the launch of vegan fast food chains with provocative names (e.g. ‘The Temple of Seitan’ in London, UK) and a recurring visual trope of plants set within a ‘bloody’ scene being chopped up by a cleaver-wielding chef or butcher (most often a white, hyper-masculine male) (see Figures 2 and 3). The promise of APs tasting, looking and performing like animal foods is thus purposefully intended to appeal to the carnivorous (typically male) consumer, one who has yet been unable to give up the sensory pleasures of animal foods and now, due to APs, does not have to.^17^

### A new paradigm?

Through the different promises identified above, the latest APs claim to break from what has gone before in terms of how animal-based foods are conceived and produced, and what can be delivered from animal-free alternatives. While specific properties of animal-based foods have been retained by APs (e.g. their sensory qualities), the notion of *disrupting* the status quo has been fundamental to AP imaginaries. Echoing the popular ideology of Silicon Valley that champions technology as a disruptive force for societal good (Morozov, 2013; Turner, 2006), the technofix approach of APs has been similarly framed by their advocates as emblematic of a new and improved paradigm in protein production and consumption. The promises of kinder, healthier, fairer, tastier, safer and more sustainable approaches to conventional livestock products thus collectively work to make the ultimate promise of a better food system for all, and in turn a better food future for all.

## Biting back: Counter-narratives from the livestock sector

In formulating this overarching promise, conventional livestock systems have typically been framed as outdated, broken and even cruel in AP narratives. In the face of these characterisations, coupled with the rapid growth in AP sales (Ridler, 2017), various stakeholders from across the conventional meat, dairy and egg industries have begun to defend their approaches, often through media interviews but also via blogs, social media and public campaigns. Such responses have come from small to large-scale farmers practising a variety of production methods, as well as industry lobby groups, governmental personnel and multinational corporations. It is important to stress this heterogeneity and note that our use of the terms ‘livestock industry/sector’ in the following section is not meant to suggest a singular entity or voice but is done for ease of reading. A range of counter-narratives has emerged which counteract, and in some cases appropriate, the hype and world-saving messaging that the recent AP movement has cultivated. The following section begins the work of mapping these responses, organising the narratives under three overarching themes.

The first counter-narrative we identify is ‘*Not a serious threat*’. Perhaps unsurprisingly, this response featured more in the earlier stages of AP development when many of the ventures and technologies that have since gone on to lead the movement were still in relative infancy. The perception of APs as non-threatening to conventional livestock industries has typically operated at two levels: first, scepticism over the technological capabilities of APs being able to produce competitive and satisfying products; and second, anticipation of consumer rejection of the particular technoscientific methods and end products of APs. An example of the former view is an online news article published in New Zealand in 2014 (RNZ, 2014). Entitled ‘Non-dairy milk not aimed at NZ’, the article details the recent formation of a California-based company (Muufri, now known as Perfect Day Foods) intending to make milk from yeast. A comment follows from Fonterra, New Zealand's largest dairy company with revenues totalling around NZ$17.2 billion in 2016. The company's response is to dismiss Perfect Day Foods as not a serious competitor to the nation's dairy sector, and to question the technical viability of their yeast-based approach in achieving nutritional equivalence with animal milk, stating that ‘it would require genetic modification and even then it [is] unlikely that artificial milk could match dairy’.

The characterisation of APs as artificial and synthetic in comparison with conventional animal foods has also been a consistent response by many stakeholders in livestock production. Such framings have reflected the view of many in the livestock industry that APs will not be an appealing alternative for consumers, and instead remain in the category of ‘Frankenfood’. However, with the rapid growth of AP sales over the last decade, livestock advocates have begun to deploy these negative characterisations in a more active and defamatory way. This has resulted in a second counter-narrative category – ‘*Not real food*’ – which seeks to challenge the ‘clean’ image cultivated by the AP sector through highlighting the techno-scientific nature of its production – specifically the movement's use of biomedical techniques, laboratories and in some cases genetic engineering.

An extreme manifestation of this counter-narrative made headlines on both sides of the Atlantic in 2015 when it was revealed that the American Egg Board (AEB) was involved in a campaign to counteract the growing popularity of JUST's plant-based egg products (Thielman, 2015). Through an USDA-led investigation, it was discovered that a central strategy of this campaign was to promote the ‘realness’ of chicken-laid eggs in direct contrast to alternative products (USDA, 2016). Echoes of this narrative can be found on AEB's website on a page entitled ‘REAL Eggs or Egg Replacers?’:

Accept no substitutes! Over the past few years there's been a great deal of discussion, research and application work done to replace eggs with various products. And while any number of companies are working hard to develop a product that can compete head on with this most versatile of ingredients – the fact of the matter is – when eggs are added, it simply appears on the ingredient statement as eggs. On the other hand, adding replacers increases the complexity of your ingredient statement.

The challenge set against APs here echoes the rules for good eating advocated by popular food writer Michael Pollan, amongst which he suggests that we should not eat products with more than five ingredients, nor ingredients that you cannot pronounce (Pollan, 2008). This counter-narrative accuses APs of adding complexity and even deceit to food-consumer relations, as well as raising unnecessary and/or unfounded health implications (Roberts, 2016). In the UK, the battle of real versus fake foods has manifested most recently in the discussion of ‘ultra-processed’ products (Boseley, 2018), a moniker which the latest APs have not escaped (Blythman, 2018). For many involved in slower, local and organic food movements, particularly those relating to livestock, the ultra-processed nature of APs signals the latest case of Big Food going too far in its industrialised substitution of whole, real foods (Biltekoff, 2016; Guthman, 2015), and a further step away from cultivating more localised systems of care and trust (Blythman, 2018; Boseley, 2018; Roberts, 2016).^18^

In part related to these battles over the qualification of real food, a third counter-narrative has struck at the heart of an unfolding ontological and regulatory conflict concerning the labelling of AP products (AFP, 2018). We term this ‘*Not legally defined*’. At contest here is the question of what can and should be legally classified under the labels of meat, milk and other animal-food terminology. Such disputes have worked across two characteristics of animal and animal-free products: (a) provenance, that is the raw materials and location of production; and (b) production methods, for example conventional livestock rearing, tissue engineering and so on. Livestock industry lobby groups have been amongst the most vocal in these disputes. In the US, the Cattlemen's Association (USCA) recently filed a petition calling on the Food Safety and Inspection Service (FSIS) – an agency of the United States Department of Agriculture (USDA) – to sharpen the regulatory definition of meat, and to do so in such a way that directly excludes APs:

…products that are labeled as ‘meat’ should be limited to those that are derived from the tissue or flesh of an animal harvested in the traditional manner. As such, USCA requests that FSIS exclude man-made or artificially manufactured products that are not derived from animals born, raised, and harvested in the traditional manner from the definition of both beef and meat. This includes synthetic products from plant, insects, and other nonanimal components, as well as any product grown in labs from animal cells. (USCA, 2018: 2)

Similarly, emphasising the importance of conventional provenance and production methods, the National Cattlemen's Beef Association (NCBA) has called for tighter regulations on labelling products as beef, stating that ‘beef should only be applicable to products derived from actual livestock raised by farmers and ranchers’ (NCBA, 2018: 1).

Missouri has become the first US state to officially sanction these interpretations; passed in May 2018, the bill defines meat as ‘any edible portion of livestock or poultry carcass or part thereof’ (General Assembly of the State of Missouri, 2018: 17) and prohibits individuals from ‘misrepresenting a product as meat that is not derived from harvested production livestock or poultry’ (General Assembly of the State of Missouri, 2018: 24). Outside the US, France recently made headlines for passing a bill that similarly reinforces the link between conventional livestock rearing and meat-related nomenclature. The reasons given by Jean-Baptiste Moreau, a member of parliament and farmer, reflect those underpinning the US disputes: to counteract the ‘false claims’ of animal-food analogues and thereby eliminate consumer confusion; to restore the ‘true value’ of agricultural products; and to increase access to ‘healthier and more sustainable food’ (cited in Askew, 2018).

It is not only the definition of meat that has been subject to such debates: across North America and Europe, consumer protection groups and dairy industry representatives have been engaged in a long contest over the use of labels such as butter and milk to describe animal-free alternatives (BBC, 2017; Plant Based News, 2017). A civil lawsuit was also filed in 2014 by Unilever, owners of Hellman's Mayonnaise, against JUST for their use of the term ‘mayo’ to describe their plant-based products. The multinational company claimed JUST's use of the label was misleading customers and unfairly stealing market share (Kowitt, 2014). Considerable consumer backlash subsequently led Unilever to drop the lawsuit.

Such debates look set to continue as AP advocates have launched a number of counteractions, arguing that the use of animal-based terminology is the most appropriate for products such as cultured meat and milk given their biological equivalence, and that consumer deception can be sufficiently minimised through clear labelling of the alternative ingredients (AFP, 2018). Moreover, there has also been pushback from *within* the livestock sector against the ‘*Not legally defined*’ counter-narrative: in its defence of classifying meat as the product of an animal carcass, the NCBA has stated that ‘lab-grown meat’ products should be included within ‘the statutory definition of a [*sic*] meat food products’ (NCBA, 2018: 2). This statement challenges USCA's view (at the time of writing) that cultured meat should be disqualified from this category along with plant-based and insect products. The rationale given by NCBA is to create a level playing field within the market: ‘If producers of lab-grown or cultured meat products wish to call these products meat, they must adhere to the same stringent food safety inspection standards and comply with the same set of labelling mandates as all other traditional meat food products’ (NCBA, 2018: 3).^19^ Furthermore, other major players in the livestock sector have actively incorporated APs into their portfolios: Tyson Foods and Cargill are amongst the most high-profile companies to have directly invested in AP ventures, describing these products as promising market opportunities for broadening consumer choice and complementing their conventional livestock operations (Peters, 2017; Shieber, 2018).

## Contested framings, contested futures

Both the promises of AP developers and the counter-narratives of the conventional livestock sector reveal a set of contested visions over what qualifies as a better protein-food system. This narrative battleground centres on a collection of anxieties and hopes concerning the welfare of people, animals and the planet, both in the present day and in the future, as well as appeals to the pleasure and socioeconomic value associated with animal-based foods. This is another iteration of the longstanding dispute over the meaning of good food (Guthman, 2003; Johnston, 2008; Mol, 2009), and over the types of questions *about* food that are perceived to matter by different stakeholders (Biltekoff, 2016). These debates also touch on yet broader issues relating to the contested place of science, technology and capitalism in the ordering of postmodern societies.

**Figure 4.**
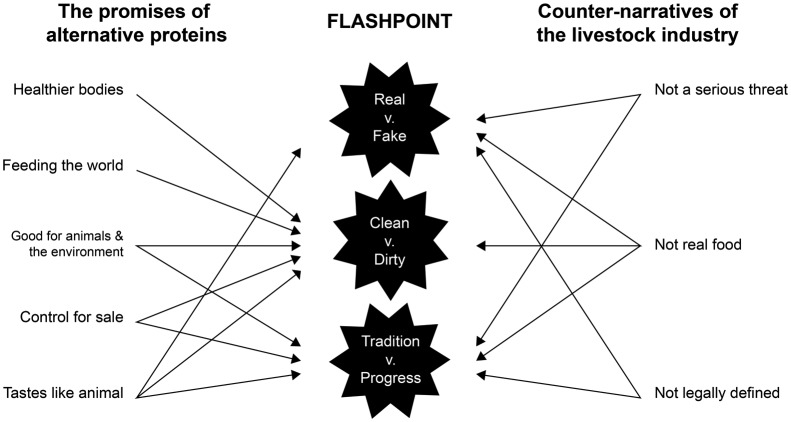
Figure identifying the three flashpoints (centre column) between the promissory narratives of alternative proteins (left) and the counter-narratives of the livestock industry (right).

Yet if we cut the narratives in a different way we can go further in discerning what exactly is being contested in these claims of goodness. Through the range of AP promises and the livestock counter-narratives we document, three distinct yet interrelated binaries emerge: ‘real vs. fake’, ‘clean vs. dirty’ and ‘tradition vs. progress’ (Figure 4). These highly politicised and emotive binaries have long characterised broader debates on food and eating (Biltekoff, 2016; Hasnain, 2018; Stanziani, 2008), striking at the heart of concerns over what food ‘is’, what it should be, and what it is becoming in the modern era under increasing industrialisation and globalisation (Goodman et al., 1987; McMichael, 2009).

What we find in these narratives, then, are attempts by the AP movement and livestock sector to position themselves as more favourable – and the other as less so – within these established binaries. In many instances, we found that notions of goodness were not exclusive to only one pole of each binary: for example being clean *and* dirty, and emblematic of tradition *and* progress, has simultaneously served to promote both AP and livestock products as the better option, albeit in different ways. The assignment of goodness to the binary of ‘real vs. fake’ food was, however, mutually exclusive – there were no instances we found where being perceived as fake food was encouraged in the framings of either AP or conventional livestock stakeholders.

Notions of the realness and cleanliness of APs were typically linked to their provenance and production methods, which in turn translated to the materiality of their end products. For cellular agriculture and plant-based APs, their realness was assured by claims that they are *technically* meat, milk and eggs at the molecular level (cf. Sexton, 2018), and as such offered equivalent nutritional content and sensory properties despite their origins outside animal bodies. As Mark Post described at the 2013 cultured beef-burger launch in London, ‘by our technology we are producing meat, it's just not in a cow’. For insect products, their claims of being real food have similarly focussed on their nutritional equivalence, but also call upon the broader histories of insect consumption (entomophagy) in non-Western contexts. Their realness is in part established by emphasising the longevity of entomophagy as a food practice; in doing so, insects are situated within the more familiar and romanticised framing of ‘food culture’. They are thus food for the foodie as well as for the adventurer/gym-goer seeking a ‘complete protein source’.

The cleanliness of APs has been most explicitly evoked in the recent coining of the term ‘clean meat’. According to advocates, this phraseology serves two purposes: it is considered a more accurate and appealing replacement specifically for the term ‘cultured meat’, and it is seen as ‘immediately communicat[ing] important aspects of the technology—both the environmental benefits and the decrease in food-borne pathogens and drug residues’ (Friedrich, 2016). The cleanliness of APs is thus delivered in two ways: first, through their methods of production – that is, the relocation from the ‘dirty’ environments of animal bodies and slaughterhouses to the ‘clean’ spaces of laboratories and technoscience; and second, through the materiality of their end products, from which the bad nutrients and pathogens of conventional animal foods have been removed. As such, the cleanliness of APs works across a range of promises, from increased control to healthier bodies, in addition to offering a kinder, *morally* clean alternative that benefits animals and the environment, as well as a solution to global hunger.

Yet in cultivating this clean image, AP developers have been careful not to present their products as *too* ‘good’ to eat so that the benefits are perceived by the public as a compromise in taste and enjoyment. Central to this balancing act is the work being done by AP developers to make their products viscerally indistinguishable from their conventional counterparts, and reveals the ‘dirty’ foundations of their clean-eating promise. Rather than a choice of abstinence and worthiness, APs instead offer a tasty and hedonistic way of saving the world. Making plants bleed and cells sizzle retains a degree of animalness – and arguably macho-ness – which in turn offers the pretence of defilement that is deeply associated with the consumption of animal flesh, as well as other animal-derived products (Douglas, 1966; Vialles, 1994). The blood is retained but not spilled, neither literally from animal carcasses nor metaphorically through harm done to human wellbeing and the planet. APs thus offer a seemingly perfect balance between clean/dirty eating, framed as the ultimate guilt-free guilty-pleasure that purportedly achieves the meaning of good food more fully than any previous animal-food analogue.

In the counter-narratives of the livestock industry, the claims that APs constitute real food were made entirely unacceptable on account of their provenance and production methods. Key to this framing was the different use or overall absence of animals from AP production. Fundamental to the livestock sector's appeal to the realness of their products has been the direct connection with living animal bodies – to appropriate Mark Post's quote above, their products are meat, milk and eggs very much produced *in* animals, and this corporeal relationship is the basis upon which realness has been asserted. Underpinning this argument is the necessity of living animal bodies having done labour (milk, eggs) and in some cases animal bodies having died (meat), the latter of which we have shown is currently upheld in regulatory definitions of meat in certain countries.

The link between animal bodies and the land, as well as the people who reared them, has also been fundamental to the livestock sector's defence of the realness of their products, as well as to notions of cleanliness *and* dirtiness in different ways. The importance placed on these connections is salient in the debates over the classification of meat, with groups such as the NCBA explicitly referencing ‘actual livestock raised by farmers and ranchers’ as the necessary conditions for labelling products as beef. Evoking the idealised imaginaries of bucolic livelihoods and of *terroir* – a concept often used by artisanal producers to capture the significance of place in the taste and quality of a foodstuff (Feagan, 2007) – has served to promote a sense of greater naturalness, wholesomeness and correctness that APs have seemingly perverted. Goodness is evoked in the direct connection between animal bodies and the ‘good dirt’ of natural landscapes, which in turn provides an evocative binary to the sterile and Frankenstein imaginaries of AP methods. This wholesome, natural dirt was credited as providing the pleasurable taste and texture of conventional livestock products, yet also evoked a sense of purity that has long been associated with romanticised visions of unspoilt landscapes (Lorimer, 2015). Embedded within these appeals to the natural is thus a distinction between the bad dirt of urbanised and techno-industrial spaces – to which APs ostensibly belong – and the good or ‘clean’ dirt of rural imaginaries. This distinction is apparent in the counter-narratives of both big and small livestock producers, regardless of whether bucolic landscapes are indeed an everyday reality of their production. With terms such as clean meat implicitly positioning conventional livestock products as dirty, the particular cleanliness promised from the wholesome natures of conventional animal agriculture thus works to (re)frame APs as clean in unnatural and untrustworthy ways.

These idealised networks of animals, people and the land have also been framed as providing a *simpler* system of protein production. A central part of the livestock sector's promotion of animal foods as the ‘real’ choice has been to position them as the less tampered with and thus more natural and trustworthy option compared with the highly processed natures of APs. This framing goes in direct contrast to the strategies that have previously characterised the narratives of intensive agriculture, in which industrial intervention has been justified as de-risking unruly animal natures and thereby improving quality and safety (e.g. Paxson, 2008). Instead, what we find in the livestock counter-narratives against APs, including those from Big Food giants and lobby groups, are echoes of the ‘artisanal reaction’ that has previously been used against proponents of industrial farming and food technofixes, such as GMOs (Murdoch and Miele, 2004). The accusations of placelessness and the loss of quality and transparency that have long been levelled at intensive agriculture are now being (re)directed to the AP movement, and are coming from both big and small livestock producers. This signals a potential evolution in the defendants of the ‘quality turn’ in food production (Murdoch et al., 2000), a trend we highlight for further analytical attention by food researchers.

The balance between retaining traditional values and offering a leap in technological/societal progress speaks to the third and final binary we identify in the AP and livestock narratives. For APs, connection to the ‘old’ ways of conventional livestock production was most explicitly delivered through the visceral mimicry of their end products; in doing so, the nostalgia for the taste and cultural significance of popular animal-based dishes, such as the ‘good ol’ fashioned burger’, is purposefully retained. Yet as noted earlier, the notion of *disrupting* the status quo is fundamental to AP imaginaries, and in the same manner as Big Tech ideologies in other sectors (Morozov, 2013; Turner, 2006), this disruption is perceived as emblematic of a new and improved paradigm for individual and societal good. This is apparent across the promises of kinder, healthier, more controlled and tastier approaches, all of which are credited directly to the AP movement's turn to the methods and innovation models of Big Tech and bioscience.

While AP narratives have appealed to hopes of change through techno-salvation, the livestock counter-narratives instead emphasised the continuance of traditional values and practices. It is a sense of tradition that underpins their appeals to realness, whereby real food is connected to the preservation of current pastoral landscapes and livelihoods, and corporeally connected with animal natures. Again, this was most explicitly communicated in the ongoing debates over the legal classifications of animal-based foods, in which the defence of conventional provenance and production methods has become bound up in the defence of preserving the true value of agricultural products and livelihoods.

Where (technological) innovation was referenced in the counter-narratives, its role was typically assigned to aiding the continuance of conventional methods. Notions of progress in the livestock counter-narratives were less a paradigm shift than improvements *within* the status quo, through which the rural imaginaries and conventional methods of production are purposefully maintained.

## Conclusion

This paper offers a critical examination of the narrative landscape that has emerged with the recent AP movement. Building on recent studies that have concentrated on specific AP products (e.g. Jönsson, 2016; Mouat and Prince, 2018; Stephens and Ruivenkamp, 2016), our first aim has been to offer a broader analysis of the key promises that have worked *across* the movement. While acknowledging the interconnected nature of these framings, the five-fold typology is intended as a heuristic for making sense of the distinct claims that have operated in key AP promotional discourses to date. A second aim of the paper was to conduct a similar mapping exercise of the responses such claims have triggered from a variety of conventional livestock stakeholders. To our knowledge, such an analysis has yet to be conducted within AP research. Our analysis offers a further typology of three counter-narratives that have shaped these reactions. While we use the terms ‘livestock industry/sector’ as shorthand, it is important to note the heterogeneity of voices behind these counter-narratives: the responses we analysed reflected a broad range of farm size, production methods and livestock type, as well as interests that encompassed producers, industry lobbyists, policymakers and consumer groups. The analysis also revealed instances of pushback against the counter-narratives from *within* the livestock sector, and also cases of active support and investment in APs from major players in the industry.

In mapping this narrative landscape, we have shown how different types of goodness have been ascribed by AP and conventional livestock stakeholders to their different approaches and products. Inherent to these framings, then, has on one level been an ontological contest over the meaning of *good* (protein) food for all (Mol, 2009), both in contemporary and future systems of production and consumption. Yet on another level, the narratives reveal a contest over the classification of what exactly (protein) food *is* and should be (Sexton, 2018; Stephens, 2013; Yates-Doerr 2015a). The binaries of ‘real vs. fake’, ‘clean vs. dirty’ and ‘tradition vs. progress’ that surfaced through our analysis reflect some of the key flashpoints around which this ontological politics has so far coalesced. These tensions convey a range of hopes and fears that have long characterised food-related debates (Jackson, 2015; Scholliers, 2008). They also touch on broader issues concerning the roles of technology and capitalism in its systems of production and consumption (Goodman et al., 1987), as well as the acceptability of animal life – and specifically animal *death* – in providing human sustenance (Buller and Roe, 2018).

However, this ontological struggle also reveals potentially new dynamics that break from those observed in previous research on contested food framings: for example where other studies have revealed distinct divides between the framings of industrial versus more wholesome, slow and artisanal food (e.g. Biltekoff, 2016), in the counter-narratives above we note the active appropriation by Big Livestock of framings previously used against them by smaller food producers. We do not suggest such actions are indicative of a formal alliance between these traditionally opposed poles, nor that the narratives always reflect the lived realities on the ground; however, there is an interesting overlap in the values and notions of what ‘food’ is that are being operationalised against APs across different scales and production modes of incumbent livestock stakeholders. We highlight this as a potential new evolution of the artisanal reaction (Murdoch and Miele, 2004) that requires continued analysis by food researchers.

There is much scope for continued examination of the trends we highlight, as well as consideration of what has been silenced in this narrative landscape. Analysis of the latter has not been possible within the scope of this paper, but there are important questions to ask regarding, for example, the gendered politics of APs, namely their reproduction of the long-held reification of (hyper)masculinity and meat (Adams, 1990; Fiddes, 1991). The reproduction of (Western) privilege through the AP visions of feeding the world also requires further critical reflection, as does the particular role of Big Tech in shaping ideas of how a better food system can be achieved. As we have shown, narratives have played a core part of this promissory work, and it is within this narrative space that the contest over the framing and future of animal-based foods looks set to continue.

## Highlights

- A distinct movement of high-tech alternative approaches to conventional livestock production has emerged over the last decade
- To date, these alternative proteins (APs) have been consumed more through promissory narratives than as tangible foodstuffs
- Five distinct AP ‘promises’ and three key counter-narratives from conventional livestock stakeholders are identified
- This narrative analysis reveals how different types of ‘goodness’ have been ascribed to both AP and conventional approaches
- Three key tensions underpinning these narratives are also identified which touch on broader debates over ‘good’ and ‘better’ food

## Notes

1. ‘Animal-free’ here refers to products that exclude ingredients sourced from conventional livestock typically used for food production in Western agriculture, including cattle, sheep, pigs, poultry, etc. Our inclusion of insects as an ‘animal-free alternative’ in our analysis is thus not to infer the non-animalness of insects, but rather signifies their positioning by advocates in recent years as an alternative to the more conventional animal species that have dominated Western meat production.

2. Our use of ‘protein’ in this paper reflects the multiplicity of meaning that has become common in the narratives we analyse: protein is often used interchangeably to denote a nutritional category, a biochemical unit, and a food group in its own right (e.g. replacing ‘meat’ for ‘protein’ in the context of describing a meal).

3. Non-edible products such as cultured leather are also included in the category of cellular agriculture (see www.new-harvest.org/about).

4. For example, the Good Food Institute's 2018 conference in San Francisco, CA, entitled ‘The future of meat: Accelerating plant-based and clean meat industries’, and their recently-launched funding grant for ‘accelerat[ing] the development of plant-based and clean meat products’ (GFI, 2018).

5. The term ‘single-cell protein’ refers to ‘microbial protein … produced from bacteria, yeasts, microscopic fungi, and microscopic algae’ that can be used for human or animal food (Mateles and Tannenbaum, 1968: 42).

6. Exceptions to this trend within the plant-based AP companies include JUST, Beyond Meat and most recently Impossible Foods who have expanded their products across a range of low to high-cost retailers and restaurants both within and increasingly outside the US; yet when considered on a global scale, the lower price points still remain unaffordable for many populations around the world.

7. The rise of protein as a global concern is a trend with a longer history that exceeds the scope of this paper; for discussion of this, see Carpenter (1986).

8. All figures on funding rounds retrieved in May 2018 from www.crunchbase.com.

9. For other qualitative studies on the corporate websites of (alternative) food brands, see PAD Research Group (2016) and Fuentes and Fuentes (2017).

10. In 2013, the first cultured meat burger was unveiled to the public, an event that is frequently perceived as a formative moment in bringing wide-reaching public attention to AP activity (Stephens and Ruivenkamp, 2016).

11. These time periods were chosen due to the concentration of key AP events resulting in high-profile coverage across global media: for example the cultured beef burger tasting event in 2013, and the series of FDA and USDA meetings on the regulatory considerations of APs during 2018.

12. Where quotation marks appear in this paper without a direct literary reference (e.g. [Name] [Year]), they denote direct quotations taken from the AP narrative materials we analysed.

13. The figures 16% and 21% are lifted from the following section of Sinha et al.’s (2009: 567) paper: ‘For overall mortality, 11% of deaths in men and 16% of deaths in women could be prevented if people decreased their red meat consumption to the level of intake in the first quintile. The impact on CVD [cardiovascular disease] mortality was an 11% decrease in men and a 21% decrease in women if the red meat consumption was decreased to the amount consumed by individuals in the first quintile’. It appears the AP company in question has selected the highest mortality figures (i.e. women) for each of the categories; it also appears that the figure of 16% has been interpreted by the company as mortality risk due to cancer specifically, whereas the original study includes this figure under the different analytical category of ‘overall mortality’ (with ‘cancer mortality’ and ‘CVD mortality’ forming the other two categories).

14. The precise weight and production methods of their own products and the conventional livestock being assessed were not given.

15. An asterisk shows that the figures represent a comparison between the company's alternative burger and, in the company's words, ‘a typical US beef production system’.

16. A possible reason for this bias was raised during the first author's fieldwork interviews: a number of AP companies stated that improved animal welfare is a key benefit of their approaches, but that market data suggests consumer choice is more strongly influenced by messaging on health and the environment. The choice of some companies to not emphasise animal welfare or use labels such as ‘vegan’ has also aimed to broaden their target audiences beyond solely plant-based eaters.

17. For recent and ongoing work on masculinites, meat and protein, see The ‘Man Food Project’ (https://man-food.org/).

18. The launch of social media campaign ‘Februdairy’ by UK farmers – a direct response to the ‘Veganuary’ movement – is another example of the defence of ‘real’ dairy over animal-free alternatives (Howell, 2018).

19. Within the same document, however, they also petition the USDA to prevent cultured meat from being advertised as ‘beef’ and to not allow ‘arbitrary marketing claims’ and ‘misleading labels like “clean meat”’.
